# Mood disorders as a risk factor for family aggregation of somatic diseases

**DOI:** 10.1192/j.eurpsy.2022.1426

**Published:** 2022-09-01

**Authors:** E. Kasyanov, G. Mazo

**Affiliations:** Bekhterev National Medical Center for Psychiatry and Neurology, Translational Psychiatry, Saint-Petersburg , Russian Federation

**Keywords:** Depression, somatic diseases, family study, bipolar disorder

## Abstract

**Introduction:**

Mood disorders (MDs) are associated with somatic diseases and tend to aggregate in families. But there are limited studies on the risk of somatic diseases for relatives of patients with MDs.

**Objectives:**

To assess whether a patient’s mood disorder diagnosis is associated with a family history of somatic disorders.

**Methods:**

This cross-sectional family study included 36 patients with MDs (66.7% women; age - 32 [11.2] years) and 68 of their relatives, and 23 healthy individuals (56,5% women; age - 30.5 [6.9] years) and 53 of their relatives. A Pearson’s χ2 test was used to compare the frequencies of family history of somatic disease. Logistic regression models were used to determine the independent association of MDs, after adjusting for the effects of sex, age, with binary characteristics.

**Results:**

Individuals with and without MDs had different frequencies of family history of cardiovascular (66,7% vs. 43,4%; p=0,03) and endocrinological diseases (47,2% vs. 39,1%; p=0,04). There were no statistically significant differences in the frequency of family history of gastrointestinal, pulmonary, urogenital and musculoskeletal diseases (p>0,05). Logistic regression revealed that MDs diagnosis in patients was a risk factor for cardiovascular (p=0.03, OR=3.5) and endocrinological disease (p=0.04, OR=3.7) in their relatives.

**Conclusions:**

MDs are associated with the aggregation of somatic diseases in families. Future research is needed to clarify the biological reasons for this association.

**Disclosure:**

No significant relationships.

